# The impact of modifiable health metrics on mortality for older adults with low cognitive function

**DOI:** 10.3389/fpubh.2024.1304876

**Published:** 2024-01-25

**Authors:** Wei Wang, Pengfei Sun, Tingting Lv, Min Li

**Affiliations:** ^1^Department of Clinical Laboratory, Beijing Friendship Hospital, Capital Medical University, Beijing, China; ^2^Department of Ultrasound, Beijing Friendship Hospital, Capital Medical University, Beijing, China; ^3^Liver Center, Beijing Friendship Hospital, Capital Medical University, Beijing, China; ^4^Clinical Epidemiology and EBM Unit, Beijing Friendship Hospital, Beijing Clinical Research Institute, Capital Medical University, Beijing, China

**Keywords:** cognition, mortality, aged, exercise, healthy diet

## Abstract

**Objectives:**

Cognitive impairment has emerged as a major contributing factor to mortality for older adults. Identifying the strong modifiable health metrics against mortality is of high priority, especially in this high-risk population.

**Methods:**

This population-based study used data of US adults aged≥60 years old from the National Health and Nutrition Examination Survey 2011–2014 cycles. De-identified data for participants who completed cognitive function test were extracted. Mortality data was obtained by linking to the 2019 public-use linked mortality file.

**Results:**

Participants with low global cognition had higher risk of all-cause mortality (HR = 1.46; 95%CI, 1.04–2.05). The highest prevalence of ideal level of health metrics was observed for sleep duration (54.36% vs. 62.37%), and the lowest was noted for blood pressure (12.06% vs. 21.25%) for participants with low and average to high global cognition, respectively. Ideal status of physical activity and diet quality were significantly associated with all-cause mortality among participants with low global cognition (HR = 0.48, 95%CI: 0.28–0.82; HR = 0.63, 95%CI: 0.43–0.95). The corresponding population-attributable fractions were 26.58 and 15.90%, respectively.

**Conclusion:**

Low cognitive function was associated with increased risk of all-cause death for older adults. Attainment of healthy metrics, especially sufficient physical activity, consuming healthy diet and being never smoked, provided strong protection against death risk.

## Introduction

Longer life expectancy has increasing the proportion of older adults and leads to a commensurate growing prevalence of cognitive impairment worldwide (1). Cognitive impairment, characterized by subtle changes in memory and thinking, is a common phenomenon in older adults and can be seen as a prodrome of Alzheimer disease (2). Recent data suggests that the global prevalence of cognitive impairment is approximately 12%–18% for people aged more than 60 years older, causing an enormous economic burden (3).

Even at mild level, cognitive impairment is linked to an increased mortality risk in older adults (4, 5). Although vascular risk factors and comorbidities might explain the increased mortality associated with cognitive impairment to some extent, cognitive impairment itself has independent implication for mortality throughout the aging process (6). Therefore, it is important to identify the modifiable health metrics as well as the stronger protection ones against all-cause mortality for individuals with cognitive impairment. It is well documented that health behaviors, such as sufficient physical activity and healthy diet, could promote healthy aging and enhance quality of life in adults (7, 8), but the evidence among older adults with cognitive impairment is lacking. Understanding the association between modifiable health metrics and mortality in older adults with cognitive impairment will stimulate further efforts to design and implement intervention strategies.

Herein, we used a nationally representative sample of the United States (US) older adults to assess the impact of modifiable health metrics on mortality, and further estimate the prevented proportion of mortality by reaching ideal health metrics for older adults with cognitive impairment.

## Materials and methods

### Study design and population

The National Health and Nutrition Examination Survey (NHANES) is a continuous, national survey to estimate the prevalence of and risk factors for major diseases. The NHANES utilizes a complex, multistage, probability sampling design that provides representative samples of the non-institutionalized US resident population (9). The NHANES procedures and protocols were approved by the National Center for Health Statistics Research Ethics Review Board. All participants provided written informed consent.

This study is based on an analysis of data from two NHANES cycles (2011–2012, 2013–2014). De-identified data for participants aged ≥60 years old, who had complete data on cognitive function, were extracted. Mortality status and cause of death were identified by linking to publicly accessible mortality files (10). All-cause, cardiovascular (CV) and cancer specific mortality was ascertained based on the underlying causes of death from the International Classification of Diseases codes. The follow-up started from the interview date and ended at death or at the end of the study (December 31, 2019).

### Definitions of low global cognition

Cognitive function was assessed for participants aged≥60 years old in NHANES 2011–2012 and 2013–2014 cycles using Consortium to Establish a Registry for Alzheimer’s Disease test (CERAD), animal fluency (AF) and digit-symbol substitution test (DSST).

The CERAD Word Learning test is a measure of immediate and delayed recall memory, using a list of 10 unrelated words. The immediate learning part consisted of three consecutive tests, where participants were requested to read the word list and recall as many as possible. This process was repeated three times with the order of words changed (11). In delayed recall part, participants were requested to recall the 10 words after approximately 8–10 min (12). The AF test is a measure of verbal fluency, where participants were asked to name as many animals as possible in 1 min, with the total score ranging from 0 to 40 (12). The DSST Test, a measure of processing speed, sustained attention and working memory, in which participants were asked to draw as many symbols paired with numbers as possible within 2 min, and the score is the total number of correct matches (13, 14).

The scores of each cognitive test were Z-score transformed. The overall cognitive score was calculated as the average of the standardized scores of the three cognitive tests. Because there was no well-defined standard regarding the threshold for identifying low cognitive performance, we selected participants with an overall cognitive score in the lowest quartile (the 25^th^ percentile) to indicate low global cognition (15–17). Meanwhile, participants with an overall cognitive score higher than that at the 25^th^ percentile were defined as having average to high global cognition.

### Definitions of modifiable health metrics

In this study, modifiable health metrics included six health behaviors (physical activity, diet quality, smoking and alcohol drinking status, sleep duration, and body mass index) and three health factors (total cholesterol, blood pressure and blood glycemic index). To capture more detailed information, modifiable health metrics were first classified into multiple levels in prevalence description, then defined as dichotomized variables with ideal and no ideal status (intermediate or poor) in risk assessment.

According to the NHANES guidelines, physical activity from the household and transportation was defined as moderate activity (18). The minutes of vigorous activity were equal to the doubled minutes of moderate activity (19). In this study, the total amount of physical activity was calculated as the minutes of equivalent moderate activity per week of all domains, and further divided into 3 levels: 0, 0.1 to 149.9, 150 min/wk. or greater (20, 21). Diet quality was assessed by the Healthy Eating Index 2015 (HEI-2015), which is a measure of dietary adherence to the 2015–2020 Americans Dietary Guidelines (22, 23). The HEI-2015 score equal or greater than 60.0 was recognized as consuming healthy diet (24). Smoking status was categorized into 3 groups: never smoked (less than 100 cigarettes per lifetime), former smoker (more than 100 cigarettes per lifetime but has quit), current smoker (more than 100 cigarettes per lifetime and currently are still smoking). Alcohol drinker was defined as had at least 12 drinks of any type of alcoholic beverage in any 1 year. Sleep duration was categorized into 3 levels: less than 7, 7 to 8, more than 8 h. Body mass index was calculated as weight in kilograms divided by height in meters squared and categorized into 3 levels: 18.5 to 24.9, 25.0 to 29.9, 30.0 kg/m^2^ or greater.

In addition, blood pressure was categorized as ideal (untreated systolic blood pressure (SBP) < 120 mmHg, diastolic blood pressure (DBP) < 80 mmHg), intermediate (SBP 120–139 mmHg, DBP 80–89 mmHg or treated), and poor (SBP ≥ 140 mmHg, DBP ≥ 90 mmHg). Total serum cholesterol was divided into 3 levels: untreated cholesterol≤200 mg/dL, 200–239 mg/dL or treated, and ≥ 240 mg/dL. Blood glycemic index was divided by HbA1c into 3 levels: less than 5.7%, 5.7–6.4%, and 6.5% or greater.

### Covariates

A set of covariates were taken into consideration. Information on age, gender, race and ethnicity, educational level, health insurance, family income, marital status, and disease history (congestive heart failure, coronary heart disease, stroke, cancer/malignancy, diabetes, metabolic syndrome) was collected. Age was divided into 5 levels: 60–64, 65–69, 70–74, 75–79, and ≥ 80. We reported gender, race and ethnicity as listed in the NHANES dataset. Education was divided into 2 groups (high school or less, some college or higher). Health insurance was categorized into 3 groups: private insurance (any private health insurance, Medi-Gap, and single-service plan), public insurance (Medicare, Medicaid, military healthcare, Indian Health Service, state-sponsored health plan, and other government insurance), and no insurance. Family income was defined by the poverty income ratio (PIR) as low (PIR ≤ 1.3), middle (PIR 1.3–3.5), and high (PIR > 3.5). Marital status was categorized into 4 groups: married, never married, living with a partner, and other. Disease history of congestive heart failure, coronary heart disease, stroke, and cancer/malignancy was defined as an affirmative response to the question, “Ever been told you have the corresponding disease?” Diabetes was defined as self-reported medical history of diabetes, using anti-diabetic medication or a fasting glucose level ≥ 126 mg/dL. Metabolic syndrome was defined based on the Adult Treatment Panel III criteria in 2005 (25).

### Statistical analysis

For the description of demographic characteristics, continuous variables were represented by median with interquartile and categorical variables by unweighted counts with weighted percentage. Differences between groups were evaluated using chi-square test for categorical variables.

Cox proportional hazard model was performed to assess the association of low global cognition with mortality. According to the classification of confounding covariates, they were progressively added to 5 adjustment models in addition to the univariate analysis (model 1): model 2, adjusted for age and gender; model 3, adjusted for socio-demographic variables including age, gender, race and ethnicity, educational level, health insurance, family income, marital status and NHANES cycle; model 4, additionally adjusted for disease history including congestive heart failure, coronary heart disease, stroke, cancer/malignancy, diabetes, and metabolic syndrome; model 5, additionally adjusted for health behaviors including physical activity, diet quality, smoking and alcohol drinking status, sleep duration, and body mass index; model 6, additionally adjusted for health factors including blood pressure, total serum cholesterol, and glycemic index. Each of the above variables was entered in the Cox proportional hazard model as separate covariates. Further, hazard ratios (HRs) of each ideal health metric as well as the number of ideal health metrics on mortality stratified by cognitive status were estimated in model 5, with no ideal status being set as reference. The variance inflation factor (VIF) was calculated to test multicollinearity, with a value of 5 or more indicating the presence of multicollinearity. The proportion of mortality that would have been prevented by reaching ideal health metric was estimated by population-attributable fraction (PAF) (26).

According to NHANES analytic guidelines, complex sampling design and sampling weights were taken into account in our analyses to produce national estimates (27). Sampling weight was reweighted in using combined NHANES cycles: fasting subsample 4-year mobile examination center (MEC) weight = fasting subsample 2-year MEC weight/2. Statistical analyses were done using SAS version 9.4 (SAS Inc., Cary, NC, United States). All analyses were two-sided, with a *p*-value less than 0.05 as statistical significance.

## Results

### Characteristics of the participants

Of 3,632 participants aged ≥60 years old, 436 were excluded for not performing cognitive tests. We also excluded 262 participants with missing data in calculating the overall cognitive score and another 10 with expected survival period less than 6 months. Therefore, a total of 2,924 participants were enrolled in the present analysis.

Compared to those with average to high global cognition, participants with low global cognition tended to be older, less educated, having less private insurance, with lower annual household income, and were less likely to be married and Non-Hispanic White. Participants with low global cognition showed a higher prevalence of congestive heart failure, stroke, and diabetes (Table 1).

**Table 1:** Baseline characteristics of the study participants by cognitive status in the NHANES 2011–2014 cycles.

Characteristics	Low global cognition (*N* = 726)	Average to high global cognition (*N* = 2,198)	*p* value
Age			<0.01
60–64	141 (14.47) [9.70–19.43]	774 (35.36) [32.29–38.43]	
65–69	139 (13.41) [10.01–16.80]	532 (25.76) [22.97–28.55]	
70–74	135 (20.33) [16.41–24.25]	414 (18.72) [16.48–20.95]	
75–79	100 (14.41) [10.91–17.92]	212 (9.75) [8.27–11.22]	
≥80	211 (37.29) [30.47–44.10]	266 (10.42) [9.07–11.76]	
Gender			0.51
Male	397 (47.05) [42.91–51.19]	1,026 (45.29) [42.94–47.63]	
Female	329 (52.95) [48.81–57.09]	1,172 (54.71) [52.37–57.06]	
Race and ethnicity			<0.01
Mexican American	79 (6.78) [2.98–10.58]	178 (2.77) [1.54–4.01]	
Other Hispanic	117 (9.74) [5.60–13.89]	179 (2.56) [1.69–3.43]	
Non-Hispanic White	246 (60.62) [50.65–70.59]	1,148 (82.93) [79.89–85.96]	
Non-Hispanic Black	224 (17.02) [10.95–23.08]	471 (6.84) [4.81–8.87]	
Other	60 (5.84) [3.72–7.95]	222 (4.90) [3.44–6.37]	
Education level			<0.01
High school or less	543 (69.15) [63.42–74.88]	882 (32.36) [28.79–35.93]	
Some college or higher	181 (30.85) [25.12–36.58]	1,315 (67.64) [64.07–71.21]	
Health insurance			<0.01
Private insurance	281 (50.03) [43.98–56.07]	1,278 (68.33) [65.49–71.16]	
Public insurance	366 (43.75) [38.72–48.78]	751 (26.43) [23.51–29.35]	
No insurance	76 (6.22) [4.15–8.30]	163 (5.24) [3.73–6.75]	
Family income			<0.01
Low	313 (38.33) [31.55–45.10]	496 (14.28) [11.39–17.17]	
Medium	229 (41.27) [36.05–46.48]	789 (37.63) [34.15–41.11]	
High	108 (20.41) [15.49–25.33]	741 (48.09) [42.70–53.49]	
Marital status			<0.01
Married	353 (49.86) [44.35–55.38]	1,256 (64.38) [62.07–66.69]	
Never married	40 (5.62) [3.05–8.20]	126 (4.09) [2.96–5.22]	
Living with partner	18 (1.91) [0.85–2.98]	61 (2.78) [1.61–3.95]	
Other	315 (42.60) [37.32–47.88]	751 (28.75) [26.86–30.64]	
Disease condition			
Congestive heart failure	77 (11.96) [9.24–14.69]	126 (5.79) [4.21–7.37]	<0.01
Coronary heart disease	78 (11.71) [8.53–14.88]	182 (8.93) [7.04–10.83]	0.06
Stroke	83 (12.18) [7.69–16.67]	117 (5.27) [4.07–6.46]	<0.01
Cancer/malignancy	124 (22.02) [16.90–27.15]	463 (24.32) [21.21–27.44]	0.51
Diabetes	262 (34.60) [29.99–39.20]	557 (21.31) [19.02–23.59]	<0.01
Metabolic syndrome	342 (46.00) [40.33–51.68]	903 (40.36) [36.25–44.47]	0.14

Data were presented as unweighted number and weighted percentage with 95% confidence interval. NHANES, National Health and Nutrition Examination Survey.

### Prevalence of modifiable health metrics

Participants with low global cognition were less engaged in physical activity, less likely to be alcohol drinkers, and had longer sleep duration as well as increased blood pressure and glycemic index. Yet, decreased total cholesterol was also observed among these participants. The highest prevalence of ideal level of health metrics was observed for regular sleep duration (54.36% vs. 62.37%), and the lowest prevalence was noted for blood pressure (12.06% vs. 21.25%) among participants with low and average to high global cognition, respectively (Table 2).

**Table 2:** Prevalence of modifiable health metrics among older adults, stratified by cognitive status.

Characteristics	Low global cognition	Average to high global cognition	*p* value
Physical activity, minutes/week			<0.01
Ideal (≥150)	269 (33.52) [27.72–39.31]	1,137 (54.42) [51.22–57.62]	
Intermediate (1–150)	122 (15.01) [11.63–18.39]	367 (15.35) [13.64–17.06]	
Poor (0)	335 (51.48) [45.94–57.02]	694 (30.23) [27.92–32.54]	
Diet quality			0.64
Ideal	208 (33.17) [28.34–38.01]	704 (34.66) [30.42–38.91]	
Poor	439 (66.83) [61.99–71.66]	1,355 (65.34) [61.09–69.58]	
Smoking status			0.52
Never	347 (48.85) [44.26–53.45]	1,093 (49.74) [46.23–53.25]	
Former	274 (38.61) [33.52–43.70]	837 (39.58) [36.50–42.65]	
Current	105 (12.54) [9.45–15.63]	266 (10.68) [9.06–12.31]	
Alcohol drinking status			<0.01
No	275 (41.69) [38.06–45.31]	638 (24.66) [21.71–27.61]	
Yes	428 (58.31) [54.69–61.94]	1,531 (75.34) [72.39–78.29]	
Sleep duration, hours/daily			<0.01
Regular (7–8)	375 (54.36) [48.37–60.35]	1,236 (62.37) [59.72–65.02]	
Short (<7)	253 (28.66) [23.77–33.54]	757 (28.11) [26.05–30.17]	
Long (>8)	97 (16.98) [13.43–20.53]	200 (9.52) [7.80–11.23]	
Body mass index			0.11
Ideal (BMI 18.5–24.9)	190 (29.34) [24.97–33.71]	552 (24.76) [21.88–27.65]	
Intermediate (BMI 25.0–29.9)	247 (37.24) [32.12–42.35]	758 (36.44) [34.19–38.69]	
Poor (BMI ≥30.0)	251 (33.43) [27.34–39.52]	831 (38.80) [35.90–41.69]	
Blood pressure, mm Hg			<0.01
Ideal (<120/80 untreated)	85 (12.06) [8.79–15.34]	330 (21.25) [18.15–24.35]	
Intermediate (120–139/80–89 or treated)	458 (70.53) [66.37–74.70]	1,249 (66.18) [62.40–69.97]	
Poor (≥140/90)	101 (17.40) [13.19–21.61]	247 (12.57) [10.52–14.61]	
Total serum cholesterol, mg/dL			<0.01
Ideal (<200 untreated)	212 (27.26) [23.20–31.32]	544 (23.79) [21.14–26.44]	
Intermediate (200–239 or treated)	434 (66.65) [62.32–70.99]	1,355 (64.33) [60.75–67.90]	
Poor (≥240)	44 (6.08) [4.08–8.09]	238 (11.88) [9.89–13.88]	
Glycemic index, HbA1c, %			<0.01
Ideal (<5.7%)	219 (34.88) [30.51–39.24]	853 (45.37) [41.78–48.96]	
Intermediate (5.7–6.4)	289 (41.04) [37.16–44.91]	899 (40.63) [37.34–43.92]	
Poor (≥6.5%)	184 (24.09) [19.26–28.92]	377 (13.99) [12.80–15.20]	
No. of ideal health metrics			<0.01
0	20 (2.69) [0.31–5.07]	42 (1.61) [0.90–2.32]	
1	96 (12.80) [9.36–16.23]	240 (9.53) [7.31–11.74]	
2	159 (21.04) [17.95–24.13]	446 (19.74) [16.94–22.54]	
3	195 (27.54) [22.78–32.30]	528 (24.95) [21.41–28.48]	
4	127 (18.94) [14.91–22.97]	462 (21.93) [19.48–24.38]	
5	70 (9.56) [6.60–12.51]	276 (12.64) [10.12–15.17]	
6	37 (4.53) [2.64–6.43]	128 (6.75) [4.64–8.86]	
7	11 (2.44) [0.34–4.55]	43 (2.30) [1.45–3.15]	
8	3 (0.46) [0.00–1.05]	7 (0.33) [0.01–0.64]	
9	0	2 (0.24) [0.00–0.57]	

Data were presented as unweighted number and weighted percentage with 95% confidence interval. BMI, body mass index.

Compared to those with average to high global cognition, participants with low global cognition tended to meet a lower number of ideal health metrics (15.49% vs. 11.14% for ≤1 ideal health metric and 16.99% vs. 22.26% for ≥5 ideal health metrics).

### Impact of cognitive function on mortality

**Figure 1:**
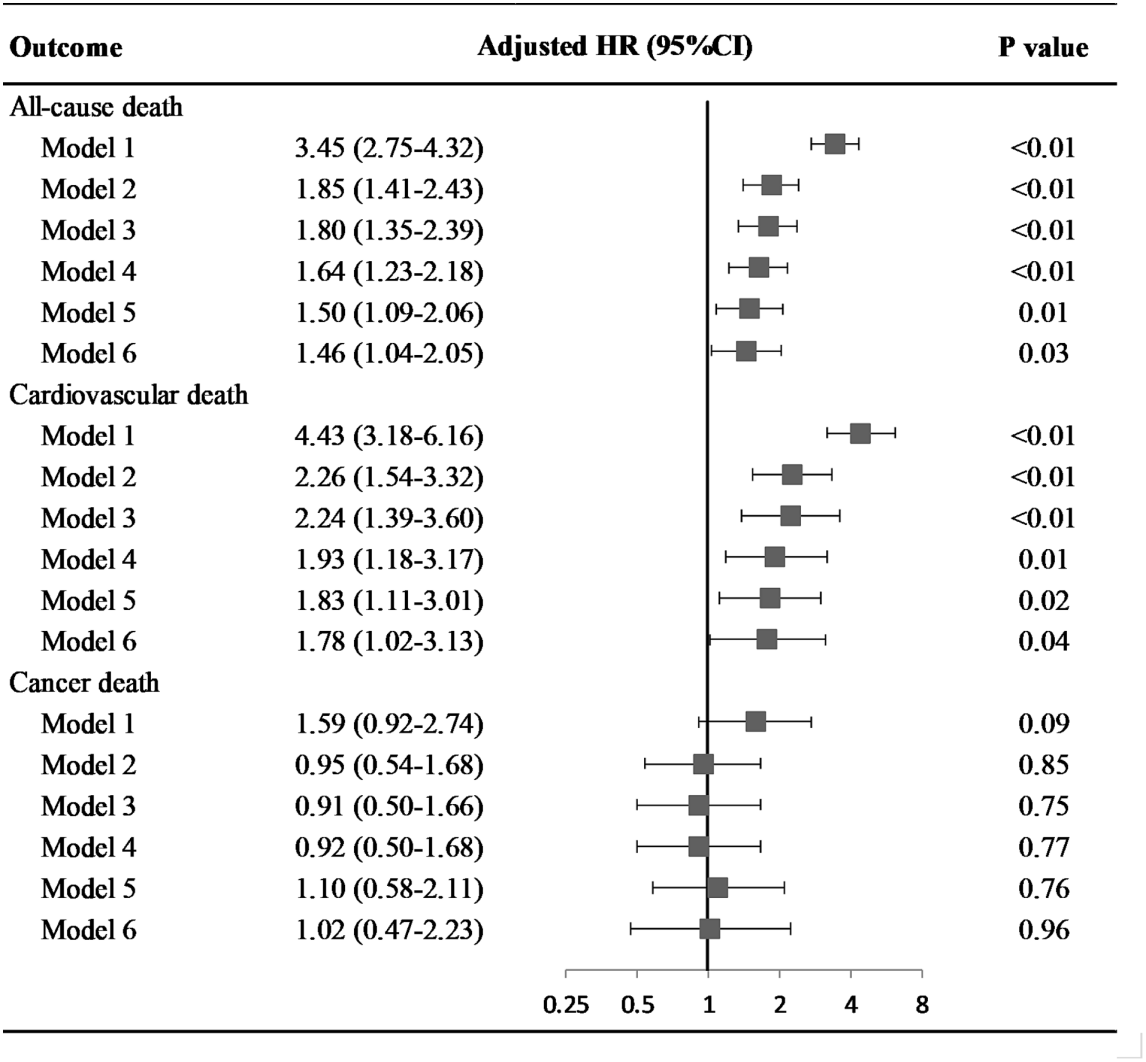
Hazard ratio of cognitive status on all-cause and disease specific death among older adults. Model 1: univariate model; Model 2: adjusted for age and gender; Model 3: adjusted for socio-demographic variables (age, gender, race and ethnicity, educational level, health insurance, family income, marital status), and NHANES cycle; Model 4: adjusted for socio-demographic variables, disease history (congestive heart failure, coronary heart disease, stroke, cancer/malignancy, diabetes, metabolic syndrome), and NHANES cycle; Model 5: adjusted for socio-demographic variables, disease history, health behaviors (physical activity, diet quality, smoking and alcohol drinking status, sleep duration, and body mass index), and NHANES cycle; Model 6: adjusted for socio-demographic variables, disease history, health behaviors, health factors (blood pressure, total serum cholesterol, and glycemic index), and NHANES cycle. Abbreviation: HR, hazard ratio; CI, confidence interval.

At a median follow-up of 6.58 years, 569 participants died (191 CV deaths, 145 cancer deaths). Participants with low global cognition had higher all-cause mortality than those with average to high global cognition (40.03% vs. 13.55%). When adjusting for a series of covariates, the association of cognitive function with all-cause mortality weakened but was still significant (HR = 1.46; 95% confidence interval, CI, 1.04–2.05) (Figure 1; Supplementary Table S1).

Increased risk of CV death was revealed in participants with low global cognition (adjusted HR = 1.78; 95%CI, 1.02–3.13). However, low global cognition was not associated with cancer death in crude and fully adjusted models, with HRs and 95%CI of 1.59 (0.92–2.74) and 1.02 (0.47–2.23) respectively.

### Impact of modifiable health metrics on mortality, stratified by cognitive status

After multivariable adjustment, ideal physical activity and diet quality were significantly associated with lower risk of all-cause death for participants with low global cognition (HR = 0.48, 95%CI: 0.28–0.82; HR = 0.63, 95%CI: 0.43–0.95). The adjusted PAFs indicated that 26.58 and 15.90% of all-cause deaths could be potentially averted by achieving the ideal level of physical activity and diet quality, respectively. Meanwhile, ideal smoking status and physical activity provided the significant protection against all-cause death for participants with average to high global cognition (HR = 0.64, 95%CI: 0.47–0.86; HR = 0.72, 95%CI: 0.52–1.00), with the adjusted PAFs of 22.09 and 17.14%, respectively. Further, with the number of ideal health metrics increased, reduced risk of all-cause death was observed for participants with average to high global cognition (*p* < 0.05). The protection was significant after achieving one ideal health metric (HR = 0.27, 95% CI: 0.09–0.85). Although protection against all-cause death was observed for participants with low global cognition, the associations were not significant (Table 3; Supplementary Table S2).

**Table 3:** Hazard ratios of modifiable health metrics on all-cause death among older adults, stratified by cognitive status.

Variables	Low global cognition		Average to high global cognition	
	Adjusted HR^*^ (95%CI)	*p*	Adjusted HR^*^ (95%CI)	*p*
Physical activity	0.48 (0.28–0.82)	0.01	0.72 (0.52–1.00)	0.05
Diet quality	0.63 (0.43–0.95)	0.03	0.93 (0.63–1.39)	0.73
Smoking status	0.91 (0.51–1.61)	0.73	0.64 (0.47–0.86)	<0.01
Alcohol drinking status	1.46 (0.87–2.45)	0.15	1.10 (0.74–1.61)	0.64
Sleep duration	0.76 (0.53–1.10)	0.14	0.94 (0.71–1.24)	0.65
Body mass index	1.64 (0.99–2.72)	0.06	0.98 (0.65–1.48)	0.93
Blood pressure	1.20 (0.63–2.27)	0.57	0.79 (0.42–1.47)	0.44
Total serum cholesterol	1.20 (0.73–2.00)	0.46	1.39 (0.94–2.05)	0.10
Glycemic index	1.19 (0.65–2.18)	0.57	0.85 (0.62–1.17)	0.30
No. of ideal health metrics				
0	Reference		Reference	
1	0.64 (0.34–1.22)	0.17	0.27 (0.09–0.85)	0.03
2	0.77 (0.44–1.34)	0.34	0.34 (0.15–0.77)	0.01
3	0.85 (0.47–1.54)	0.58	0.33 (0.12–0.91)	0.03
4	0.61 (0.33–1.12)	0.11	0.34 (0.14–0.80)	0.02
5	0.77 (0.30–1.98)	0.57	0.28 (0.10–0.75)	0.01
6	0.63 (0.29–1.40)	0.25	0.11 (0.03–0.38)	<0.01
≥7	0.32 (0.10–1.02)	0.05	0.16 (0.04–0.68)	0.01

^*^Each health metric was dichotomized (Ideal vs. no ideal), and no ideal status was set as reference. All HRs were calculated after adjusting for socio-demographic variables (age, gender, race and ethnicity, educational level, health insurance, family income, marital status), disease history (congestive heart failure, coronary heart disease, stroke, cancer/malignancy, diabetes, metabolic syndrome), health behaviors (physical activity, diet quality, smoking and alcohol drinking status, sleep duration, and body mass index), and NHANES cycle (Model 5). HR, hazard ratio; CI, confidence interval; NHANES, National Health and Nutrition Examination Survey.

Regardless of cognitive status, ideal physical activity was significantly associated with lower risk of CV death, with HRs of 0.49 (0.24–0.99) for low global cognition and 0.33 (0.17–0.64) for average to high global cognition. The corresponding PAFs for physical activity were 26.03 and 52.24%, respectively. Additionally, ideal smoking status significantly decreased the risk of CV death and contributed an adjusted PAF of 31.16% for participants with average to high global cognition. Generally, the risk of CV death decreased with increasing number of ideal health metrics for participants with average to high global cognition, but the associations were not observed for participants with low global cognition (Table 4; Supplementary Table S3).

**Table 4:** Hazard ratios of modifiable health metrics on cardiovascular death among older adults, stratified by cognitive status.

Variables	Low global cognition		Average to high global cognition	
	Adjusted HR^*^ (95%CI)	*p*	Adjusted HR^*^ (95%CI)	*p*
Physical activity	0.49 (0.24–0.99)	0.05	0.33 (0.17–0.64)	<0.01
Diet quality	0.50 (0.23–1.12)	0.09	1.29 (0.65–2.57)	0.45
Smoking status	1.83 (0.87–3.85)	0.11	0.53 (0.32–0.88)	0.02
Alcohol drinking status	1.19 (0.43–3.31)	0.73	1.78 (0.92–3.43)	0.08
Sleep duration	0.70 (0.34–1.43)	0.31	0.75 (0.39–1.45)	0.38
Body mass index	1.88 (0.93–3.80)	0.08	1.00 (0.44–2.27)	0.99
Blood pressure	1.15 (0.44–3.03)	0.77	0.68 (0.22–2.08)	0.49
Total serum cholesterol	1.35 (0.79–2.30)	0.27	0.83 (0.44–1.55)	0.54
Glycemic index	1.95 (0.65–5.82)	0.23	1.15 (0.59–2.23)	0.67
No. of ideal health metrics				
0	Reference		Reference	
1	0.90 (0.17–4.67)	0.89	0.24 (0.07–0.80)	0.02
2	0.99 (0.26–3.85)	0.99	0.25 (0.08–0.81)	0.02
3	0.99 (0.23–4.30)	0.99	0.26 (0.10–0.70)	0.01
4	0.96 (0.25–3.70)	0.95	0.20 (0.06–0.64)	0.01
5	1.02 (0.18–5.82)	0.98	0.13 (0.03–0.47)	<0.01
6	1.74 (0.43–7.13)	0.43	0.03 (0.01–0.16)	<0.01
≥7	0.50 (0.03–9.69)	0.63	0.16 (0.02–1.16)	0.07

^*^Each health metric was dichotomized (Ideal vs. no ideal), and no ideal status was set as reference. All HRs were calculated after adjusting for socio-demographic variables (age, gender, race and ethnicity, educational level, health insurance, family income, marital status), disease history (congestive heart failure, coronary heart disease, stroke, cancer/malignancy, diabetes, metabolic syndrome), health behaviors (physical activity, diet quality, smoking and alcohol drinking status, sleep duration, and body mass index), and NHANES cycle (Model 5). HR, hazard ratio; CI, confidence interval; NHANES, National Health and Nutrition Examination Survey.

Further, ideal smoking status and physical activity significantly reduced the risk of cancer death for participants with low global cognition (HR = 0.08, 95%CI: 0.01–0.49; HR = 0.12, 95%CI: 0.03–0.53). The corresponding PAFs were 84.97% and 71.19%, respectively. However, no modifiable health metric was found to be associated with cancer death among participants with average to high global cognition. Regardless of cognitive status, no association of number of ideal health metrics and risk of cancer death was observed (Table 5; Supplementary Table S4).

**Table 5:** Hazard ratios of modifiable health metrics on cancer death among older adults, stratified by cognitive status.

Variables	Low global cognition		Average to high global cognition	
	Adjusted HR^*^ (95%CI)	*p*	Adjusted HR^*^ (95%CI)	*p*
Physical activity	0.12 (0.03–0.53)	0.01	1.18 (0.67–2.07)	0.57
Diet quality	3.03 (0.87–10.65)	0.08	0.97 (0.44–2.17)	0.94
Smoking status	0.08 (0.01–0.49)	0.01	0.66 (0.34–1.28)	0.21
Alcohol drinking status	2.17 (0.39–12.26)	0.37	0.60 (0.30–1.22)	0.15
Sleep duration	2.35 (0.53–10.41)	0.25	0.78 (0.42–1.44)	0.41
Body mass index	1.64 (0.50–5.45)	0.40	0.65 (0.33–1.29)	0.21
Blood pressure	0.81 (0.11–6.09)	0.83	0.73 (0.24–2.17)	0.56
Total serum cholesterol	0.73 (0.20–2.75)	0.64	1.79 (0.76–4.23)	0.18
Glycemic index	0.55 (0.15–2.02)	0.36	0.65 (0.36–1.18)	0.15
No. of ideal health metrics				
≤1^a^	Reference		Reference	
2	0.77 (0.27–2.22)	0.62	0.88 (0.30–2.58)	0.81
3	2.81 (0.94–8.43)	0.06	0.61 (0.21–1.80)	0.36
4	1.18 (0.28–4.94)	0.81	0.86 (0.36–2.06)	0.72
5	1.20 (0.31–4.74)	0.79	1.24 (0.32–4.91)	0.75
6	0.36 (0.02–6.06)	0.47	0.39 (0.07–2.03)	0.25
≥7	0.68 (0.07–6.64)	0.73	–	–

^*^Each health metric was dichotomized (Ideal vs. no ideal), and no ideal status was set as reference. All HRs were calculated after adjusting for socio-demographic variables (age, gender, race and ethnicity, educational level, health insurance, family income, marital status), disease history (congestive heart failure, coronary heart disease, stroke, cancer/malignancy, diabetes, metabolic syndrome), health behaviors (physical activity, diet quality, smoking and alcohol drinking status, sleep duration, and body mass index), and NHANES cycle (Model 5). HR, hazard ratio; CI, confidence interval; NHANES, National Health and Nutrition Examination Survey.

a Because no cancer death was observed for older adults with low global cognition in 0 ideal metric category, adults with 0 or 1 ideal metrics were grouped as 1 category (≤1).

## Discussion

Our findings suggested that low cognitive function was associated with increased risk of all-cause death for participants aged ≥60 years old. Ideal levels of health metrics, especially sufficient physical activity, healthy diet and never smoked, were associated with significant reduction in death risk. For participants with low global cognition, nearly 27% of all-cause deaths, 26% of CV deaths, and 71% of cancer deaths could be preventable through the attainment of sufficient physical activity. In addition, consuming healthy diet could avert 16% of all-cause deaths and reporting never smoked could avert 85% of cancer deaths.

Some of previous studies indicated that the association of cognitive impairment and death risk is mainly due to the presence of well-established risk factors such as systemic vascular comorbidities (e.g., high blood pressure and diabetes mellitus) (6, 28). In our study, a higher vascular risk profile was indeed observed for individuals with low global cognition. Moreover, our data showed that low global cognition was associated with a 46% increased mortality risk in elders, regardless of the influence of classic vascular risk factors. Thus, monitoring cognition function regularly among older adults may help to identify populations at high risk of death for in-time intervention to improve prognosis. Additionally, the relation of cognitive function and disease-specific death (e.g., CV death and cancer death) may show different patterns, which need to be validated by more studies.

Studies addressing the impact of modifiable health metrics and cognitive function for the risk of mortality are scarce. This is particularly important in older adults given their increased risk of comorbidities and unhealthy metrics. In this study, our findings highlighted the importance of maintaining ideal level of health metrics for older adults. Regardless of cognitive status, sufficient physical activity provided protection against all-cause mortality. Our findings are consistent with previous studies. A population-based cohort of older adults in Spain revealed that cognitive frailty was more markedly associated with increased mortality in inactive older adults, and being active reduced the mortality risk among cognitively frail individuals by 36% (29). Another national sample of community-dwelling Taiwanese aged 65 years or older suggested that having a physically active lifestyle has beneficial effects on survival in older persons with either frailty/pre-frailty or cognitive impairment (30). Based on the available evidence, the association seems biologically plausible. Several studies suggested that physical activity could reduce the risk of death through several pathways, including increased antioxidant defenses and NO bioactivity, improved endothelial function, decreased systemic inflammation, and reduced psychosocial stress (31–33). Taking together, these evidences highlight the benefits of sufficient physical activity in reducing death risk even in older adults with cognitive impairment. Meanwhile, it is very important to pay attention to the possible bidirectional association between physical activity and cognitive function. That is, less physical activity can directly attribute to the decline of cognitive function, while the decrease in physical activity can also be the result of cognitive impairment (34, 35). Besides, an increasing body of evidence suggests that the presence of ideal health metrics during mid-life has an impact on later life cognition (36). In individuals with cognitive impairment, in general, at early stages of the disease, engaging in physical activity induces improvements in executive functions, memory, and cognitive tests (37). Thus, it is crucial to maintain the ideal health metrics for older adults to prevent and delay cognitive decline as well as lower death risk at the earliest stage possible.

Smoking status is another health metric worthy noticing. Data suggested that among participants with low global cognition, a healthy benefit against cancer death may be gained for reporting as never smoked. Meanwhile, smoking was also a large contributor to all-cause and CV death for participants with average to high global cognition, where approximately 22% and 31% of corresponding death events could be averted if the smoking status was ideal. Besides, particular attention should be paid to the cessation of smoking in either group. Although three health metrics (physical activity, diet quality and smoking status) were observed to significantly reduce the risk of death, our analysis did not find that the other six metrics, including alcohol drinking status, regular sleep duration, body mass index, blood pressure, total serum cholesterol, and glycemic index, significantly contributed to the reduction of mortality. Our results differed from previous studies on the protective effect of these six health metrics (38, 39). One explanation of the inconsistency is that these metrics may be correlated to the other three metrics such that if these three metrics are addressed, they may have an indirect effect on mortality. It has been reported that physical activity is associated with weight loss, lower blood glucose, and lower blood cholesterol. On the other hand, the benefit of certain health metrics might be underestimated after adjustment for a considerable set of confounders. Specifically, controlling for diabetes as part of the disease history covariate may make the estimate for glycemic index more conservative.

Further, our results indicated that among participants with average to high global cognition, the risk of all-cause and CV death decreased as the number of ideal health metrics increased. However, this protection was not significant for participants with low global cognition. One explanation for this may be the discrepant prevalence of each ideal health metrics depending on the cognitive status, and their different impact in lowering the risk of mortality. Given the potential benefits and that all components of these health metrics are modifiable via treatment and lifestyle modification; there is a need to improve these health metrics especially in subjects with low cognition function.

The strengths of this study included the contribution to the limited evidence examining the modifiable health metrics against mortality for older adults with cognitive impairment, and were the first to report PAFs of these modifiable factors for all-cause and disease-specific deaths. Besides, the NHANES includes a large, population-based, nationally representative sample of older US adults, which provided excellent extrapolation to the entire US population. Meanwhile, this study had some limitations. First, potential changes in cognitive function between initial measurement and death were not assessed. We were unable to determine the changes in cognitive function and its impact on mortality. Second, not all modifiable health metrics were analyzed due to the limitation of data. Third, there is possibility of misclassification of certain metrics as a result of being self-reported measures, and subjects with cognitive impairment may have more risk to self-report inaccurate data. Finally, the results were derived from participants aged ≥60 years old. It may not be extrapolated to the whole population.

## Conclusion

Low cognitive function was associated with increased risk of all-cause death for older adults, independent of classic vascular risk factors. This study highlighted that health metrics of sufficient physical activity, healthy diet and being never smoked, provided significant protection against death risk. Interventions to reinforce the modifiable health metrics to ideal levels were necessary for older adult population.

## Data availability statement

Publicly available datasets were analyzed in this study. This data can be found here: these data were derived from the following resources available in the public domain: https://www.cdc.gov/nchs/nhanes/index.htm.

## Ethics statement

The NHANES procedures and protocols were approved by the National Center for Health Statistics Research Ethics Review Board. The studies were conducted in accordance with the local legislation and institutional requirements. All participants provided written informed consent.

## Author contributions

WW: Formal analysis, Writing – original draft. PS: Formal analysis, Supervision, Writing – review & editing. TL: Funding acquisition, Supervision, Writing – review & editing. ML: Conceptualization, Funding acquisition, Supervision, Writing – review & editing.
