# Supplementary material for: The impact of modifiable health metrics on mortality for older adults with low cognitive function
Source: Front Public Health. 2024 Jan 25;12:1304876. doi: 10.3389/fpubh.2024.1304876 (PMC10850326; doi:10.3389/fpubh.2024.1304876)
Supplement: Supplementary file 1 [file Table_1.DOCX]

**The impact of** **modifiable health metrics on mortality for older adults with low cognitive function**

Wei Wang, MD ^1^, Pengfei Sun, MD ^2^, Tingting Lv, MD ^3^, Min Li, Ph.D ^4^*

**CONTENT**

**Supplemental Tables**

- **Supplemental Table 1.** The variance inflation factors of variables in fitting multivariable models
- **Supplemental Table 2.** All-cause mortality by modifiable health metrics among older adults, stratified by cognitive status
- **Supplemental Table 3.** Cardiovascular mortality by modifiable health metrics among older adults, stratified by cognitive status
- **Supplemental Table 4.** Cancer mortality by modifiable health metrics among older adults, stratified by cognitive status

Supplemental table 1. The variance inflation factors of variables in fitting multivariable models

| Variables | All-cause death | Cardiovascular death | Cancer death |
| --- | --- | --- | --- |
| Age (Reference: 60-64) |  |  |  |
| 65-69 | 1.67 | 0.99 | 1.88 |
| 70-74 | 1.40 | 1.42 | 1.72 |
| 75-79 | 1.71 | 0.98 | 1.87 |
| ≥80 | 1.43 | 1.58 | 1.44 |
| Gender | 1.10 | 1.44 | 1.37 |
| Race and ethnicity (Reference: Mexican American) | | | |
| Other Hispanic | 1.56 | 2.67 | 2.70 |
| Non-Hispanic White | 2.67 | 4.04 | 3.64 |
| Non-Hispanic Black | 2.41 | 3.27 | 2.78 |
| Other | 1.37 | 1.67 | 2.88 |
| Educational level | 1.28 | 1.44 | 1.03 |
| Health insurance (Reference: Private insurance) | | | |
| Public insurance | 1.26 | 1.37 | 0.97 |
| No insurance | 1.30 | 1.02 | 2.00 |
| Family income (Reference: Low) | | | |
| Medium | 1.18 | 1.07 | 1.60 |
| High | 2.01 | 2.15 | 1.96 |
| Missing | 1.05 | 1.12 | 1.83 |
| Marital status (Reference: Married) | | | |
| Never married | 1.15 | 1.06 | 1.49 |
| Living with partner | 0.99 | 1.06 | 1.20 |
| Other | 1.17 | 1.83 | 1.54 |
| Congestive heart failure (Reference: Yes) | | | |
| No | 1.07 | 0.88 | 1.03 |
| Unclear | 1.18 | 1.04 | 1.09 |
| Coronary heart disease (Reference: Yes) | | | |
| No | 1.22 | 1.06 | 0.85 |
| Unclear | 1.53 | 0.88 | 1.34 |
| Stroke (Reference: Yes) |  |  |  |
| No | 1.12 | 1.08 | 1.15 |
| Unclear | 1.19 | 0.87 | 1.21 |
| Cancer/malignancy (Reference: Yes) | | | |
| No | 1.01 | 0.97 | 1.11 |
| Unclear | 1.45 | 2.22 | 1.47 |
| Diabetes | 1.78 | 1.46 | 1.78 |
| Metabolic syndrome | 1.64 | 1.15 | 2.77 |
| Physical activity | 1.09 | 1.18 | 0.98 |
| Diet quality | 1.11 | 1.06 | 1.05 |
| Smoking status | 1.17 | 0.97 | 1.32 |
| Alcohol drinking status | 0.92 | 0.98 | 1.35 |
| Sleep duration | 1.05 | 1.06 | 1.22 |
| Body mass index | 1.22 | 1.16 | 1.71 |
| Blood pressure | 1.09 | 1.05 | 1.62 |
| Total serum cholesterol | 1.21 | 1.46 | 1.08 |
| Glycemic index | 1.50 | 1.22 | 0.99 |
| NHANES cycle | 0.87 | 1.00 | 0.94 |

Abbreviation: NHANES, National Health and Nutrition Examination Survey.

Supplemental table 2. All-cause mortality by modifiable health metrics among older adults, stratified by cognitive status

| Variables | Low global cognition | Average to high global cognition |
| --- | --- | --- |
| Physical activity |  |  |
| Ideal | 65 (24.81) [19.21-30.42] | 124 (10.30) (7.95-12.66) |
| No ideal | 172 (47.70) [41.98-53.43] | 208 (17.43) [14.64-20.23] |
| Diet quality |  |  |
| Ideal | 62 (36.45) [27.63-45.27] | 93 (12.61) [8.66-16.56] |
| No ideal | 147 (39.84) [33.72-45.96] | 215 (13.59) [10.83-16.36] |
| Smoking status |  |  |
| Ideal | 111 (40.94) [34.32-47.57] | 125 (10.11) [7.80-12.42] |
| No ideal | 126 (39.16) [30.40-47.91] | 207 (16.97) [13.22-20.72] |
| Alcohol drinking status |  |  |
| Ideal | 99 (45.20) [37.89-52.50] | 90 (14.27) [9.68-18.87] |
| No ideal | 131 (35.66) [28.90-42.41] | 236 (13.12) [10.35-15.90] |
| Sleep duration |  |  |
| Ideal | 125 (39.94) [33.75-46.13] | 180 (13.10) [10.48-15.71] |
| No ideal | 112 (40.20) [32.59-47.81] | 150 (14.33) [11.49-17.17] |
| Body mass index |  |  |
| Ideal | 69 (44.50) [36.97-52.03] | 89 (12.85) [8.57-17.13] |
| No ideal | 141 (35.66) [30.14-41.17] | 226 (13.17) [10.90-15.44] |
| Blood pressure |  |  |
| Ideal | 27 (44.40) [29.03-59.78] | 33 (9.04) [5.25-12.83] |
| No ideal | 169 (38.56) [32.45-44.68] | 247 (14.08) [11.86-16.29] |
| Total serum cholesterol |  |  |
| Ideal | 70 (43.46) [35.33-51.60] | 96 (16.80) [13.54-20.06] |
| No ideal | 151 (37.69) [31.50-43.88] | 230 (12.54) [9.95-15.13] |
| Glycemic index |  |  |
| Ideal | 77 (42.88) [35.15-50.62] | 120 (11.49) [8.56-14.42] |
| No ideal | 141 (36.70) [28.64-44.76] | 202 (15.27) [12.61-17.93] |

Data were presented as unweighted number and weighted percentage with 95% confidence interval;

Supplemental table 3. Cardiovascular mortality by modifiable health metrics among older adults, stratified by cognitive status

| Variables | Low global cognition | Average to high global cognition |
| --- | --- | --- |
| Physical activity |  |  |
| Ideal | 27 (10.13) [6.61-13.64] | 32 (2.36) [1.32-3.39] |
| No ideal | 65 (18.78) [13.20-24.37] | 67 (6.40) [4.27-8.53] |
| Diet quality |  |  |
| Ideal | 26 (16.51) [9.17-23.85] | 25 (3.49) [1.42-5.55] |
| No ideal | 58 (15.82) [11.10-20.53] | 67 (4.52) [3.24-5.80] |
| Smoking status |  |  |
| Ideal | 52 (19.32) [12.68-25.97] | 37 (2.66) [1.78-3.54] |
| No ideal | 40 (12.59) [7.33-17.85] | 62 (5.73) [3.76-7.71] |
| Alcohol drinking status |  |  |
| Ideal | 41 (17.80) [10.55-25.05] | 28 (5.34) [2.96-7.71] |
| No ideal | 49 (14.62) [9.41-19.83] | 70 (3.87) [2.50-5.24] |
| Sleep duration |  |  |
| Ideal | 48 (15.44) [9.85-21.03] | 53 (3.89) [2.43-5.34] |
| No ideal | 44 (16.44) [10.26-22.61] | 46 (4.75) [3.40-6.11] |
| Body mass index |  |  |
| Ideal | 31 (19.35) [11.82-26.88] | 27 (3.92) [1.53-6.31] |
| No ideal | 52 (13.18) [9.43-16.92] | 66 (4.10) [2.95-5.25] |
| Blood pressure |  |  |
| Ideal | 11 (19.23) [7.32-31.14] | 10 (3.38) [0.91-5.85] |
| No ideal | 68 (16.21) [11.92-20.51] | 68 (3.83) [2.53-5.13] |
| Total serum cholesterol |  |  |
| Ideal | 29 (16.09) [9.47-22.71] | 25 (3.81) [2.40-5.21] |
| No ideal | 57 (14.93) [10.21-19.66] | 73 (4.34) [3.05-5.63] |
| Glycemic index |  |  |
| Ideal | 28 (16.91) [10.59-23.22] | 40 (3.51) [1.84-5.18] |
| No ideal | 59 (14.51) [8.83-20.18] | 58 (4.83) [3.28-6.37] |

Data were presented as unweighted number and weighted percentage with 95% confidence interval;

Supplemental table 4. Cancer mortality by modifiable health metrics among older adults, stratified by cognitive status

| Variables | Low global cognition | Average to high global cognition |
| --- | --- | --- |
| Physical activity |  |  |
| Ideal | 15 (3.83) [1.92-5.73] | 45 (4.65) [3.18-6.13] |
| No ideal | 31 (7.62) [4.48-10.76] | 54 (4.68) [3.25-6.11] |
| Diet quality |  |  |
| Ideal | 14 (7.83) [3.07-12.59] | 27 (4.64) [2.34-6.94] |
| No ideal | 29 (6.27) [2.97-9.57] | 62 (4.39) [2.63-6.16] |
| Smoking status |  |  |
| Ideal | 14 (4.50) [2.63-6.36] | 40 (3.81) [2.44-5.18] |
| No ideal | 32 (8.11) [4.07-12.16] | 59 (5.51) [3.26-7.76] |
| Alcohol drinking status |  |  |
| Ideal | 14 (6.44) [2.83-10.04] | 25 (3.45) [1.73-5.18] |
| No ideal | 30 (6.08) [2.99-9.18] | 70 (4.79) [3.30-6.28] |
| Sleep duration |  |  |
| Ideal | 26 (7.56) [3.85-11.27] | 52 (4.53) [3.24-5.81] |
| No ideal | 20 (4.91) [1.74-8.07] | 45 (4.84) [2.69-6.99] |
| Body mass index |  |  |
| Ideal | 12 (7.06) [1.94-12.17] | 21 (3.55) [1.68-5.42] |
| No ideal | 29 (6.05) [3.70-8.40] | 73 (4.96) [3.55-6.38] |
| Blood pressure |  |  |
| Ideal | 8 (9.40) [0.77-18.02] | 12 (3.95) [1.22-6.68] |
| No ideal | 28 (5.11) [2.62-7.61] | 79 (5.18) [4.01-6.36] |
| Total serum cholesterol |  |  |
| Ideal | 12 (6.67) [1.63-11.71] | 31 (6.83) [4.18-9.47] |
| No ideal | 28 (5.93) [3.24-8.63] | 65 (3.95) [2.26-5.63] |
| Glycemic index |  |  |
| Ideal | 17 (6.36) [2.27-10.45] | 38 (4.32) [2.90- 5.74] |
| No ideal | 22 (5.87) [3.26-8.47] | 57 (4.99) [3.15-6.83] |

Data were presented as unweighted number and weighted percentage with 95% confidence interval;
